# Using the Image Analysis Method for Describing Soil Detachment by a Single Water Drop Impact

**DOI:** 10.3390/s120911527

**Published:** 2012-08-24

**Authors:** Magdalena Ryżak, Andrzej Bieganowski

**Affiliations:** Institute of Agrophysics, Polish Academy of Sciences, Doświadczalna 4, 20-290 Lublin, Poland; E-Mail: a.bieganowski@ipan.lublin.pl

**Keywords:** soil detachment measurement, splash, image analysis, single water drop

## Abstract

The aim of the present work was to develop a method based on image analysis for describing soil detachment caused by the impact of a single water drop. The method consisted of recording tracks made by splashed particles on blotting paper under an optical microscope. The analysis facilitated division of the recorded particle tracks on the paper into drops, “comets” and single particles. Additionally, the following relationships were determined: (i) the distances of splash; (ii) the surface areas of splash tracks into relation to distance; (iii) the surface areas of the solid phase transported over a given distance; and (iv) the ratio of the solid phase to the splash track area in relation to distance. Furthermore, the proposed method allowed estimation of the weight of soil transported by a single water drop splash in relation to the distance of the water drop impact. It was concluded that the method of image analysis of splashed particles facilitated analysing the results at very low water drop energy and generated by single water drops.

## 1. Introduction

Soil, a natural product of the top layer of the Earth's crust, serves multiple important functions in ecosystems. Thanks to its retention capacity, soil plays a significant role in the water cycle in Nature; it also is a landscape-shaping element and a basis for crop production [1–3]. Soil is exposed to various factors that can cause its degradation [4–6].

Erosion is a physical degradation process affecting the soil surface. This process affects not only the environment, but also the productivity and profitability of agriculture. Therefore, understanding the mechanisms of erosion and preventing it is important for agriculture and the economy. Many factors induce erosion, including water or wind activity and it can be further enhanced by inappropriate agrotechnical practices. Due to large economic and natural losses caused by erosion, the problem has been investigated by many research teams worldwide [7–10].

The splash is the first stage of water erosion. The splash erosion can be characterised as two subprocesses: detachment of the particle from the soil surface and the transport of these particles in random directions [11]. Splash experiments may be performed either in laboratory or field conditions. A vast majority of investigations are carried out in laboratories, as this provides reproducible conditions under which measurements can be performed regardless of precipitation [12]. Various types of droplet formation systems [13–15] or rainfall simulators [16] are used for producing water drops.

The measurements conducted by different researchers on single water drops can be divided into two methods:

- High speed photography or cine-photography measurements. There are reports on use of this method for e.g., (i) determination of the relationship between splash angles and soil shear strength [17]; (ii) analysis of the shape and rate of formation of the splash and corona walls depending on the surface where it has taken place [18]; and (iii) description of the splashes of dry sand [19].
- Weight measurements involve weighing the collected material transported by splash, and this includes the vast majority of investigations. Such research in various laboratories has revealed relationships between: (i) the splash weight and matric potential of a soil sample, and the soil strength [13]; (ii) splash distance and soil shear strength, particle size, and thickness of the water film on the surface of the sample [20]; and (iii) soil crusting and splash magnitude [16].

While reviewing other investigations carried out on the impact a single water drop, studies connected with soil strength on soil detachability and threshold energy [21,22] or relationships between shear strength of compacted soil and the single water drop impact [15] were also examined. In these studies, generally all splashed soil material was collected into various types of compartments, which were usually fragments or quarter sections of a circle with compartments located at varied distances [11,18].

Since the weight of the collected material must be sufficiently large for splash descriptions based on weight measurements, multiple repetitions of the splash by ejecting many successive drops are indispensable for credible weighing results. For instance, Mouzai and Bouhadef [11] used 50 mL of water ejected as single water drops for one measurement series. Therefore, the results were averages, as it was impossible to estimate the weight transported with each water drop impact. It is obvious that successive water drops changed the state of the soil surface by increasing its water content and altering its geometry (mutual arrangement of soil particles). Therefore, a method that will facilitate estimation of the weight of soil transported by a single water drop splash should be developed. Given the sensitivity and resolution of laboratory scales, such a method cannot rely on weight measurements.

The aim of this study was to develop a method for describing soil detachment caused by a single water drop impact based on image analysis of tracks of splashed particles recorded on blotting paper. This method should give scientists involved in studying erosion new possibilities for measuring splash caused by a single drop of water. This was previously impossible by using weight measurements.

## 2. Experimental Section

The experiments were conducted on soil sampled from two arable soil profiles. The characteristics of the soil material are summarized in Table 1. The particle size distribution (PSD) of the soil samples (sieved through a 2 mm mesh) was determined by laser diffraction method using a Malvern Mastersizer 2000 (Malvern, UK) diffractometer. The measurements were performed using a Hydro G dispersion unit with the pump speed of 1,750 r.p.m. and stirrer speed of 700 r.p.m. [23]. PSD was determined with the use of Mie's theory [24], assuming the following values of the indices: refraction index 1.52 and absorption index 0.1 for the dispersed phase, and refraction index of 1.33 for water as the dispersing phase [25]. The obscuration was established between 10 and 20%. If the obscuration during the dispersion process (35 W ultrasonification probe during a time interval of 4 min) increased over the upper limit of 20%, the soil suspension was diluted according to the procedure described by Bieganowski *et al.* [26].

In order to examine the splashes, air-dry soil samples (sieved through a 2 mm mesh) were wetted with water, thoroughly stirred, and placed in aluminium rings of height 1 cm and diameter 3.6 cm. The rings were secured with a chiffon cloth at the bottom in order to prevent soil loss. The samples were then placed in standard pressure chambers (Soil Moisture Equipment Corp., Santa Barbara, CA, USA), where at the pressure of 16 kJ·m^−3^ (15.6 kPa) they reached field capacity corresponding to pF 2.2. At this pF value, the gravimetric water content was 23% for soil 553 and 21% for soil 590. For each soil, 15 rings were prepared, wetted and then placed in a specially prepared hole in the table. The ring filled the drilled hole tightly and the soil level (*i.e.*, the top surface of the ring) was level with the table top. The table top was then covered with a sheet of blotting paper with a central aperture cut out. The size of the aperture was large enough to prevent the falling droplets from hitting the edge of the blotting paper, and small enough for the splashed soil particles to fall on the blotting paper rather than on the soil surface. The size of the blotting paper was selected to be large enough to collect all splashed particles.

**Figure 1.**
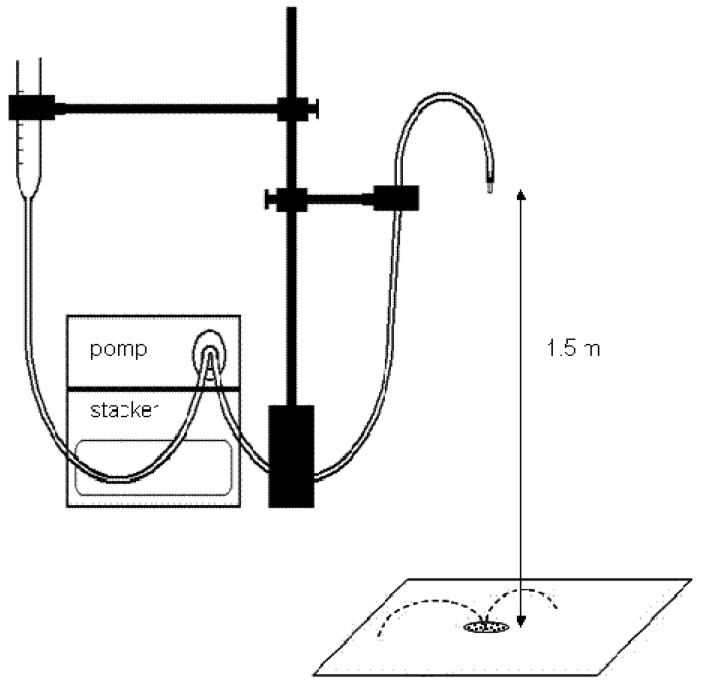
The scheme of the measurement system.

The droplet-forming system (Figure 1) consisted of: (a) an Aqua-trend Series 100 Micro Peristaltic Pump with a flow rate of 0.96 mL/min, coupled with a device controlling single or continuous droplet dispensing; (b) a water container connected with the pump by water inlet hoses; (c) a hose transporting water from the pump to the pipette, from which water drops detached freely under gravitational force; and (d) a stand for precise ejection of droplets onto a defined site from a defined height. Droplets with a diameter of 4.18 mm falling freely from a height of 1.5 m were used for the measurements.

Ten water drops were ejected onto each soil sample at time intervals sufficient to record particles that were splashed upon each water drop impact. When the splashed soil particles fell on the blotting paper, the wet tracks were numbered and the distance from the site of waterdrop impact on the sample surface was measured. Next, the tracks were carefully cut out, dried and placed under a microscope for further analysis.

**Figure 2.**
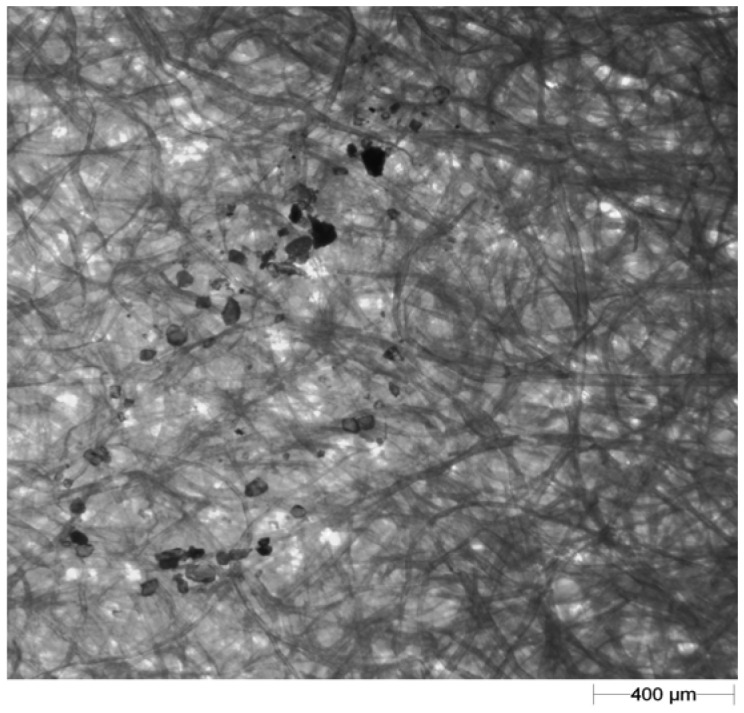
An example image of splashed particles on blotting paper.

A light microscope (Morphology G3; Malvern Instruments Ltd., Malvern, UK) was employed for image recording. A 2.5× objective lens with 123× magnification was used. In order to prevent deposition of dust particles on the blotting paper during image recording [28], scanning of the blotting paper bearing splash tracks (the shape created by the soil particles after splash) was performed in a room equipped with air filters. An example of the images of particles splashed on the blotting paper recorded under the microscope is shown in Figure 2.

Since the splashed particles were collected on blotting paper (whose texture does not allow a direct image analysis), it was necessary to establish an additional procedure for image analysis in order to distinguish the soil particles from blotting paper fibres in the recorded microscopic images. Filtration of the recorded images was performed using the Matlab R2011a (MathWorks, Natick, MA, USA) program.

**Figure 3.**
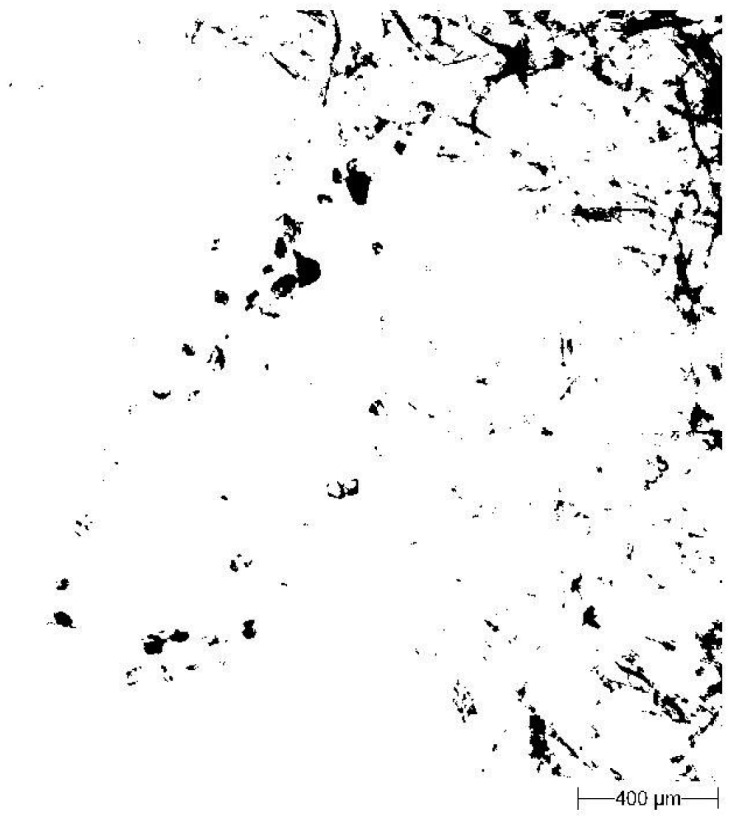
The same soil particles as Figure 2 after filtration of the blotting paper texture at the 0.32 grey level.

**Figure 4.**
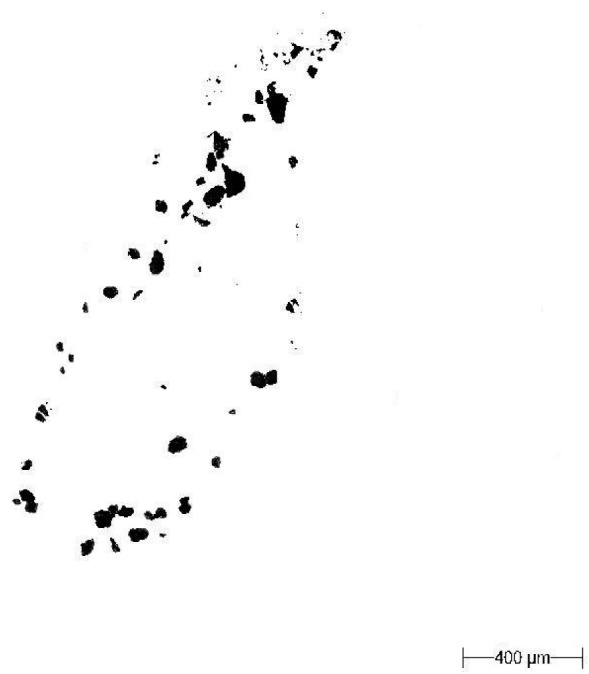
The same soil particles as Figures 2 and 3 after correction in the GIMP program.

The analysis of the microscopic images consisted of the following steps:

- Filtration of images by a specially suited grey level in Matlab (Figure 3);
- A light microscope (Morphology G3; Malvern Instruments Ltd.) was employed for image correction of the images in the GIMP program (Figure 4);
- Calculation of the parameters of the particle size and shape *i.e.*, particle surface area, CE diameter (diameter of a circle with the same area as the particle), solidity (proportion of pixels in the convex hull area) and particle area.

**Figure 5.**
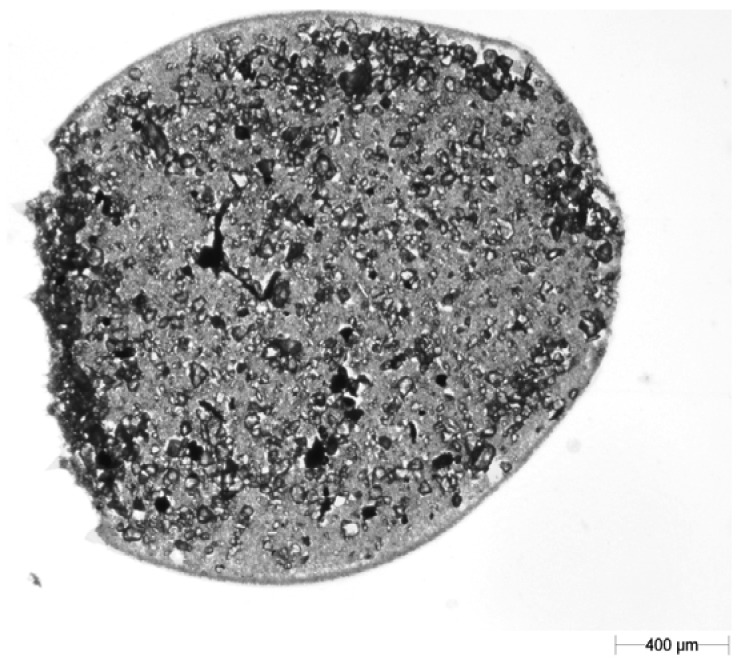
An example of an image of particles collected on the film.

Since image processing prior to analysis (*i.e.*, removal of the image of blotting paper fibres) of the shape and size parameters was time consuming and a potential source of error, an attempt was made to collect splashed soil particles on wrapping film or other kinds of papers (including coloured blue or green—colours that do not occur in soil). The film transparency or colour of the base allowed for easier identification of the splash image and suggested that the pre-processing procedure in the Matlab and GIMP programs may have been unnecessary. Unfortunately during the validation process of the wrapping film and coloured papers we could not find such material (papers or foils) sufficiently absorptive to avoid the slipping or secondary reflection from their surface—thus it was not possible to measure the distance of splashed particles. The second problem that appeared during initial attempts at using the wrapping film and other papers was the image of splashed particles. Due to the small wetting angle of the foil and to surface tension, the soil-water suspension remained drop-shaped after the splash. After evaporation of water, the soil particles were compacted so tightly that a three-dimensional lump was formed, which rendered the shape analysis pointless (Figure 5). Taking into account the abovementioned facts, analysis of particles using blotting paper was chosen. We decided to use the tedious and potentially faulty procedure described above (with Matlab and GIPM) while being aware of the possibility of using better paper in future. Use of material more suitable than blotting paper simplifies the procedure but will not change the basis of the proposed method.

## 3. Results and Discussion

**Figure 6.**
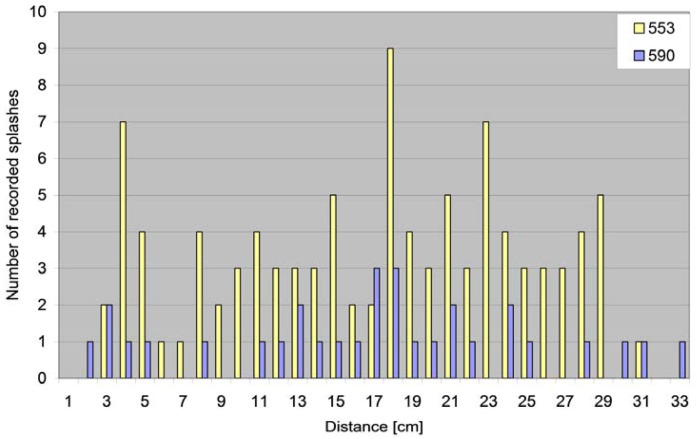
The distances of recorded splashes (distances rounded to the nearest centimetre). Because the aluminium rings with the soil samples were set horizontally (the slope of the upper sample surface was equal to zero) the splashes occurred in all directions with no preference.

By ejecting 10 drops onto each of the 15 cylinders, 100 blotting paper sections were obtained for soil 553, and 31 sections for soil 590. Each blotting paper section represented one recorded splash. The distances at which the particles were dispersed from the site of water drop impact is presented in Figure 6. An example of the ability to easily and more accurately (compared with the commonly used compartments) record splash distances is shown in Figure 6. This could be the basis of further investigations, similar to those presented by Van Dijk and co-workers [29].

**Figure 7.**
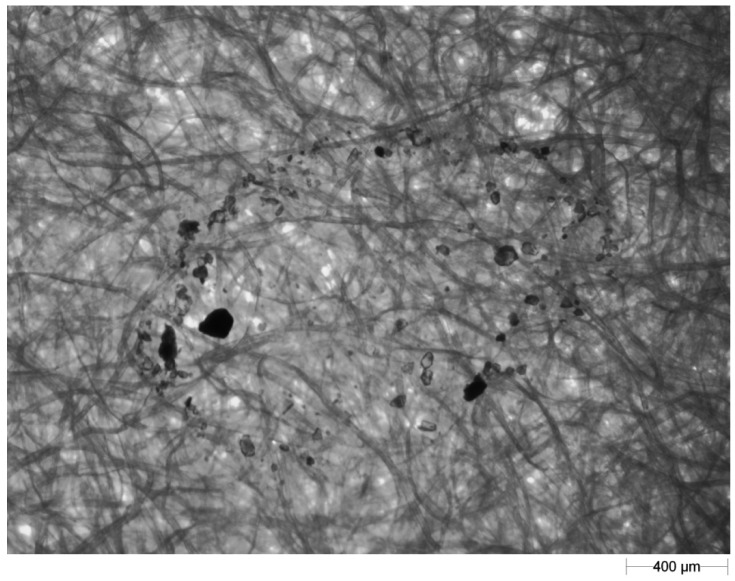
An example image of a drop recorded on blotting paper. The arrangement of all soil particles after drying allows reconstruction of the shape of the splash-inducing drop.

**Figure 8.**
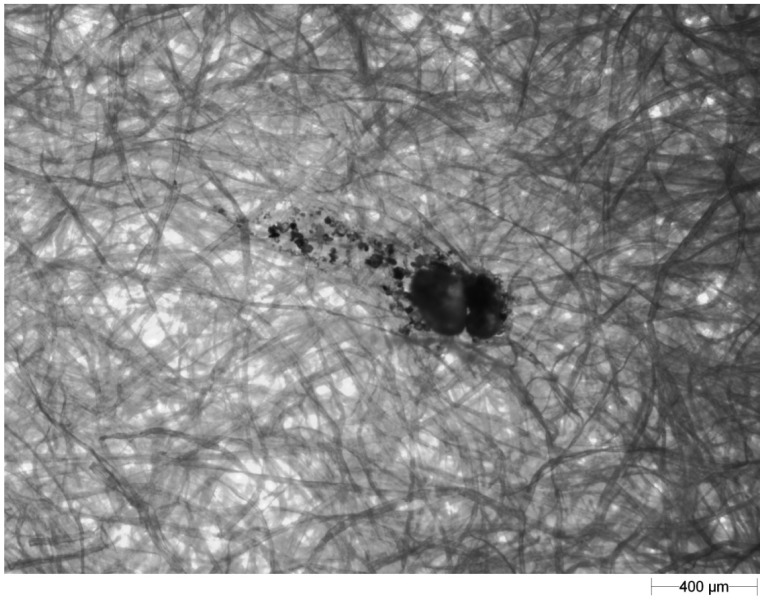
An example image of a ‘comet’ recorded on blotting paper.

**Figure 9.**
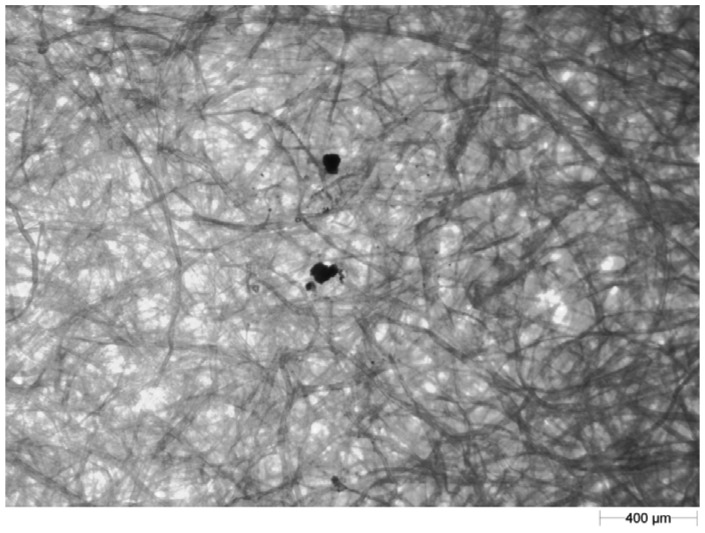
An example image of loose particles recorded on blotting paper.

Since the observed splash tracks (the shape created by the soil particles after splash) exhibited varied shapes, they were divided into three groups: droplets (e.g., Figure 7), “comets” (e.g., Figure 8) and loose particles (e.g., Figure 9).

The shape of the tracks produced by the splash on the blotting paper, *i.e.*, drops, comets or loose particles (Table 2), was probably associated with multiple factors, e.g., the energy of the drop impact and the condition of the sample surface (e.g., water content, density, PSD and presence of water film). This will be the subject of a further study.

Ghadiri and Payne [18] conducted microscopic observations of tracks left by splash drops on Perspex coated with a film of dye-drops observed ranged in size from 10 μm to 3 mm; *i.e.*, smaller than those recorded in the present study. This difference may be related to two phenomena:

- In the study of Ghadiri and Payne [18], the drops fell on a smooth surface. Therefore, secondary bouncing and/or break up of the drops into smaller ones upon impact on the Perspex surface were highly probable. Furthermore, upon impact, the drop evaporates and reduces its volume (surface area recorded under a microscope). It is difficult to estimate the scale of this phenomenon in the cited publication, as neither the laboratory conditions (temperature and humidity) nor the time elapsed between the splash and image recording were mentioned;
- The drops in our investigations fell on the hygroscopic surface of blotting paper. Thus they immediately enlarged their area, as the water was transported by capillary forces. Hence, overestimated values of the drop surface areas should be expected. However, the obvious water dispersal throughout the blotting paper was not necessarily accompanied by dispersal of soil particles, which were transported by the water, but not to the same extent as the water. Since the drop size in our study was estimated on the basis of the track of the solid phase (soil particles), it can be assumed that the error associated with hygroscopic infiltration of water into the blotting paper was negligible.

**Figure 10.**
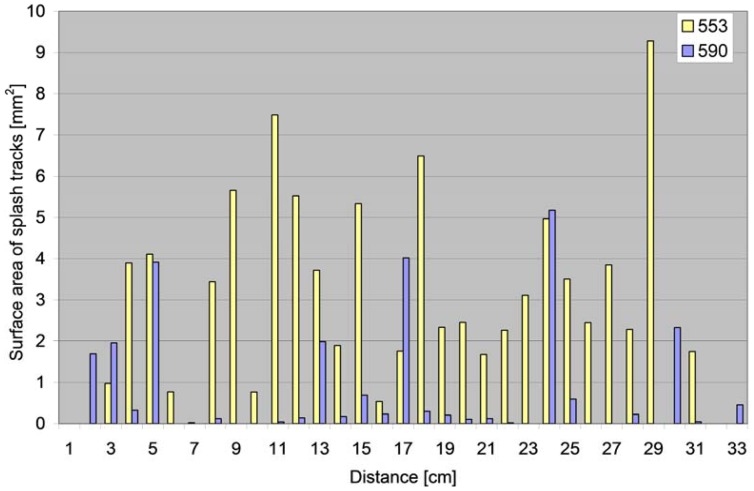
The surface areas of splash tracks recorded on the blotting paper in relation to distance (distances rounded to the nearest centimetre).

The volumes of the recorded splashes (*i.e.*, the surface areas of the drop or comet contours or single particles) in relation to the distance from the site of the water drop impact that induced the splash are presented in Figure 10.

**Figure 11.**
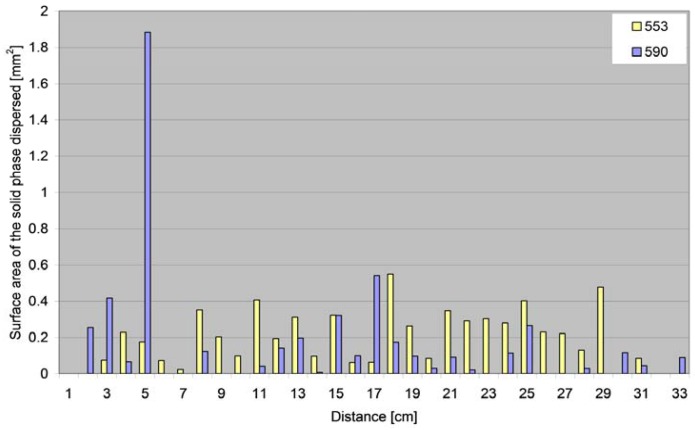
The surface areas of the solid phase dispersed at a given distance (distances rounded to the nearest centimetre).

Another important parameter determined on the basis of image analysis was the surface area of the solid phase recorded in individual blotting paper sections in relation to the distance covered by the particles (Figure 11).

The surface area is a measure of the splashed soil volume/weight; for comparison of soils, using this procedure comparing the surface areas of the solid phase particles would be sufficient. However, given that the error of such a method is extremely difficult to determine, soil weight may be estimated using the calculated surface area of the solid phase particles. To facilitate this, the volume of splashed soil particles should be measured and soil density should be assumed.

It can be assumed that the density of the solid phase for mineral soils is 2.65 g·cm^−3^, although it is more difficult to estimate their volume. A microscopic image is two-dimensional; therefore, it provides no information about particle height. Particle height may be estimated by analysing particles at the maximum magnification and the lowest lens focus depth. In this case, precise adjustment of the table (for the Morphology G3, the table can be raised and lowered by 1 μm) would allow estimation of the particle height. However, we adopted different procedure:

- the CE diameter was determined for each particle;
- based on the diameter, the density of a sphere with this diameter was calculated;
- the calculated volume and assumed density served for calculation of the weight.

In the case of loose particles (individual sand grains or individual soil aggregates) such a procedure for determination of weight does not raise major objections. However, for droplets and comet particles that are flat, the procedure would lead to overestimation of the weight. Calibration is necessary in such situations, and was carried out as follows:

- a rectangle was cut out of blotting paper on which there was a trace of a flat particle, with about 5 mm left on each side. Blotting paper was cut when particles were wet because dry soil sprang off the surface of blotting paper;
- soil traces were dried and weighed;
- a rectangle was cut out of clean blotting paper, with a similar surface area to those with soil, and weighed;
- we scanned all (with and without the soil) cut pieces of blotting paper. Image analysis software was used to calculate the surface area of the paper pieces;
- the weight and surface areas of clean blotting paper enabled calculation of the density of the blotting paper;
- the density and surface area of blotting paper with the traces of soil enabled calculation of the weight of dry soil in droplets and comets.

**Figure 12.**
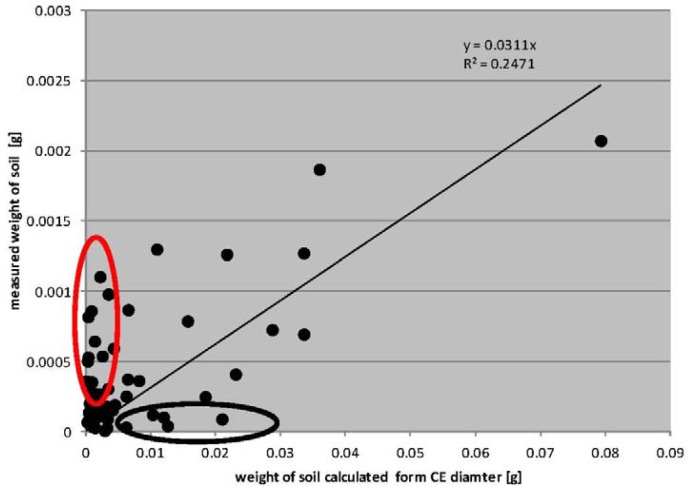
Comparison of the calculated weight on the basis of CE diameter and measured weight. The intersection of the interpolated line with the origin of the coordinate system was forced.

**Figure 13.**
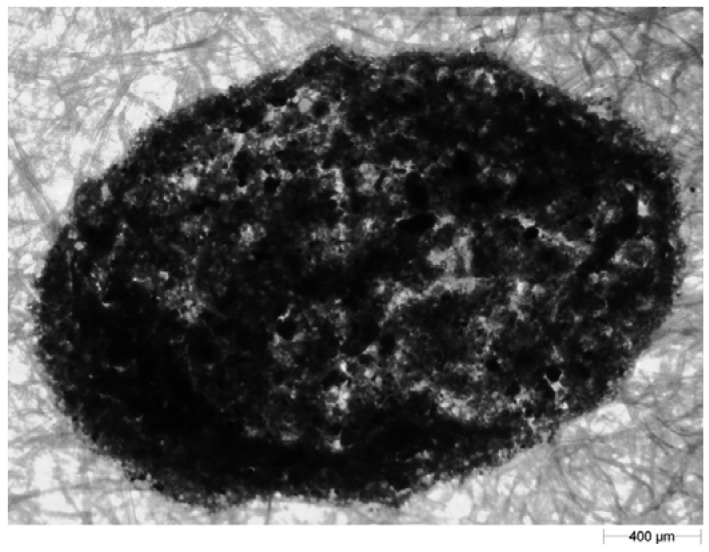
An example image of a particle with a comparatively large weight calculated from CE diameter.

**Figure 14.**
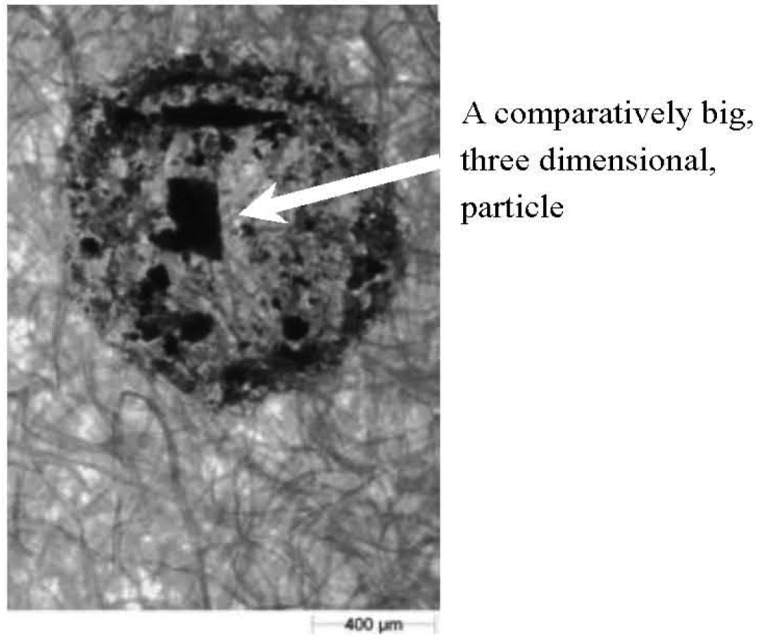
An example image of a trace with a comparatively large measured weight.

Full understanding of the information contained in the graph (Figure 12) requires a few comments:

1. The value of the determination coefficient in the graph is very small. Therefore, the cases marked by the black and red ovals should be considered;
2. The black oval–the weight calculated from CE diameter is comparatively high. There were mainly those cases where the entire surface of a trace of dried droplets was practically completely covered by the soil (Figure 13) This situation was usually occurred when the splashed soil was very wet;
3. The red oval—the measured weight was comparatively high. There were mainly those cases where only the contour of the droplet was covered by soil particles; however, inside this contour was a large (three dimensional) sand or soil aggregate (Figure 14).

**Figure 15.**
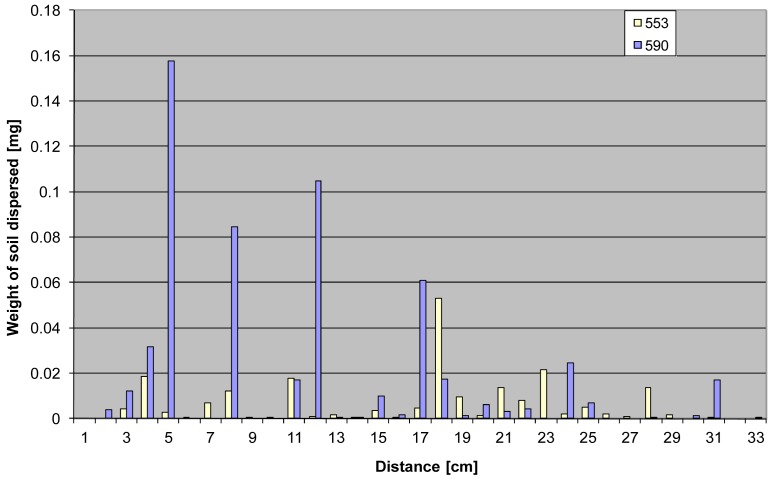
Weight of soil dispersed by splash in relation to the distance of the waterdrop impact.

Awareness of this and wanting to apply the method proposed in this paper for determining the weight of splashed soil it is necessary to calibrate for specific soils and specific moisture contents. Because we didn't prepare such a detailed calibration Figure 15 should be rather treated as the example of potential possibilities of the method. In preparing Figure 15, areas were not separated (corresponding to red and black areas) and the weight of flat traces was calculated using the equation in Figure 12.

The total weight of the splash-dispersed particles was almost threefold higher in soil 590 than in soil 553, although fewer splashes were observed for soil 590 than for soil 553 so we can stated that the method is valid for comparison of soils and suitable for the quantitative description of the phenomenon.

It is difficult to verify these data with the literature, as we found no publications on the weight of the solid phase particles for particular single water drop splashes. Al-Durrah and Bradford [13] weighed splashes on scales with accuracy of 0.1 mg; hence, it may be concluded that the weight values were obtained from a splash occurring upon the fall of many single drops. Nearing and Bradford [30] did not provide information about the number of single drops that had been ejected before the soil material was collected and weighed with an accuracy of 0.01 mg after drying. Mouzai and Bouhadef [15] used 50 mL of water for each measurement series, which corresponded to 834 drops; hence, there was no possibility of comparing our results with the other data. Verification of these calculations by weight method was also impossible, because the weight values were too small to obtain credible results.

**Figure 16.**
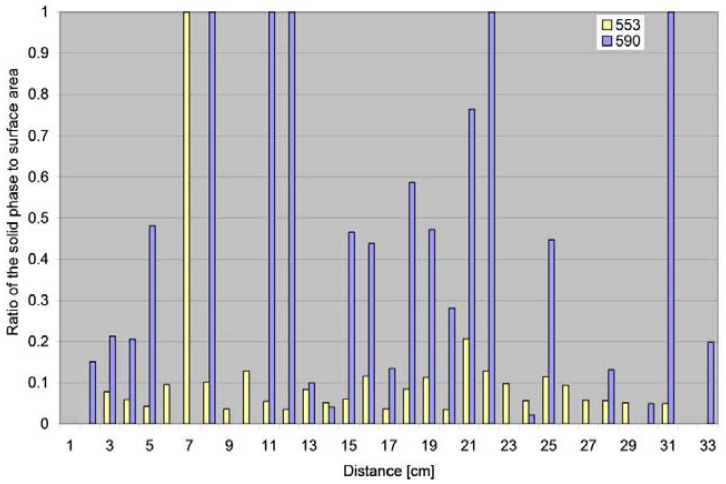
The ratio of the solid phase to the surface area of the splash track in relation to the distance (distances were rounded to the nearest centimetre).

Given the areas of droplets, comets and of the solid phase, it is possible to calculate the ratio of these surface areas. The ratios in relation to the splash distances are presented in Figure 16. In the case of soil 553, the ratio of the solid phase to the particle contour equalled 1 only for one sample, *i.e.*, particles without water tracks (dispersion at a distance of 7 cm) were only observed. In the other cases, the ratio of the solid phase to the contours of the drop or comet was lower and ≤0.2. In this soil, nearly 80% of all particles in the sample originated from the silt and clay fractions, *i.e.*, ≤0.05 mm (Table 1). The single splashed particles were also smaller in this soil (<0.3 mm; Table 2) than in soil 590. In soil 590, the ratio of the solid phase to the particle contour equalled 1 in five samples, and >0.4 in six cases. The soil was characterised by predominance of the sand fraction (>0.05 mm; Table 1); additionally, the single splashed particles were larger (up to 0.6 mm; Table 2).

## 4. Conclusions

1. The image analysis of splashed particles facilitated analysing the results at very low water drop energy and energy generated by single water drops. It excluded the necessity to average the results of many repetitions (many drops) and to average the distance (by eliminating the need to use compartments). The analysis allowed examination of even the smallest particle tracks by ascribing them to a particular water drop without the limitation of the minimum weight value that can be recorded on the scales.
2. The chosen method allowed a description of differences in splashes between different soils and/or the same soil in different states, based on a very small number of water drops and without the necessity to wait for erosive deposition.
3. Determination of the conditions of formation of drops, comets or single particles through the splash requires additional investigations to elucidate these mechanisms

## Figures and Tables

**Table 1.** Particle size distribution of the soils examined.

Soil profile number *	**Soil**		**Particle size distribution** **(%, diameter mm)**			C_org._%

	Type of soil	Granulometric group	Sand 2–0.05	Silt 0.05–0.002	Clay <0.002	
553	*Eutric Cambisol*	loamy silt	20.07	73.91	6.02	0.82
590	*Orthic Luvisol*	sandy loam	57.40	38.88	3.72	0.46

* soil sample number in the Bank of Soil Samples at the Institute of Agrophysics, Polish Academy of Sciences, Lublin [27].

**Table 2.** Characteristics of particles obtained as a result of splash.

	**Soil 553**			**Soil 590**		

Type of section	droplet	Comet	loose particles	droplet	comet	loose particles
% in populations (number of observations)	52	28	20	32	39	29
% of populations (solid phase surface)	45	45	10	21	70	9
The range of size (mm)	0.2–3.8	1.3–9.5	0.01–0.3	0.4–2.5	1.0–4.4	0.01–0.6
The average size (mm)	1.416	3.390	0.196	1.151	2.379	0.342
Standard deviation of size (mm)	0.836	2.170	0.095	0.652	1.143	0.181
Coefficient of variation	0.59	0.64	0.49	0.57	0.48	0.53
